# Supplementary material for: Interleukin 31 receptor α promotes smooth muscle cell contraction and airway hyperresponsiveness in asthma
Source: Nat Commun. 2023 Dec 11;14:8207. doi: 10.1038/s41467-023-44040-1 (PMC10713652; doi:10.1038/s41467-023-44040-1)
Supplement: Supplementary file 1 — Supplementary Information [file 41467_2023_44040_MOESM1_ESM.pdf]

Interleukin 31 receptor α promotes smooth muscle cell contraction and airway hyperresponsiveness in asthma

Santoshi Akkenepally[1,2,#], Dan JK Yombo[3,#], Sanjana Yerubandi[1,3], Geereddy Bhanuprakash Reddy[2], Deepak A. Deshpande[4], Francis X. McCormack[1], and Satish K Madala[1,*]

[1] Division of Pulmonary, Critical Care and Sleep Medicine, Department of Internal Medicine, University of Cincinnati, Cincinnati, Ohio USA.

[2] Division of Biochemistry, National Institute of Nutrition, Hyderabad, Telangana, India.

[3] Division of Pulmonary Medicine, Department of Pediatrics, Cincinnati Children's Hospital Medical Center, Cincinnati, Ohio USA.

[4] Division of Pulmonary, Allergy, and Critical Care Medicine, Center for Translational Medicine, Jane and Leonard Korman Respiratory Institute, Sidney Kimmel Medical College, Thomas Jefferson University, Philadelphia, Pennsylvania.

[#] These authors contributed equally: Santoshi Akkenepally and Dan JK Yombo

*** Correspondence:** Satish K. Madala, Division of Pulmonary, Critical Care and Sleep Medicine, The University of Cincinnati, 231 Albert Sabin Way, Cincinnati, OH 45267. E-mail: madalash@ucmail.uc.edu

This file includes:

Supplementary Figures 1, 2, 3, 4, 5, 6, 7, 8, 9, 10, 11, 12, 13, 14 and 15.

Supplementary Tables 1 and 2

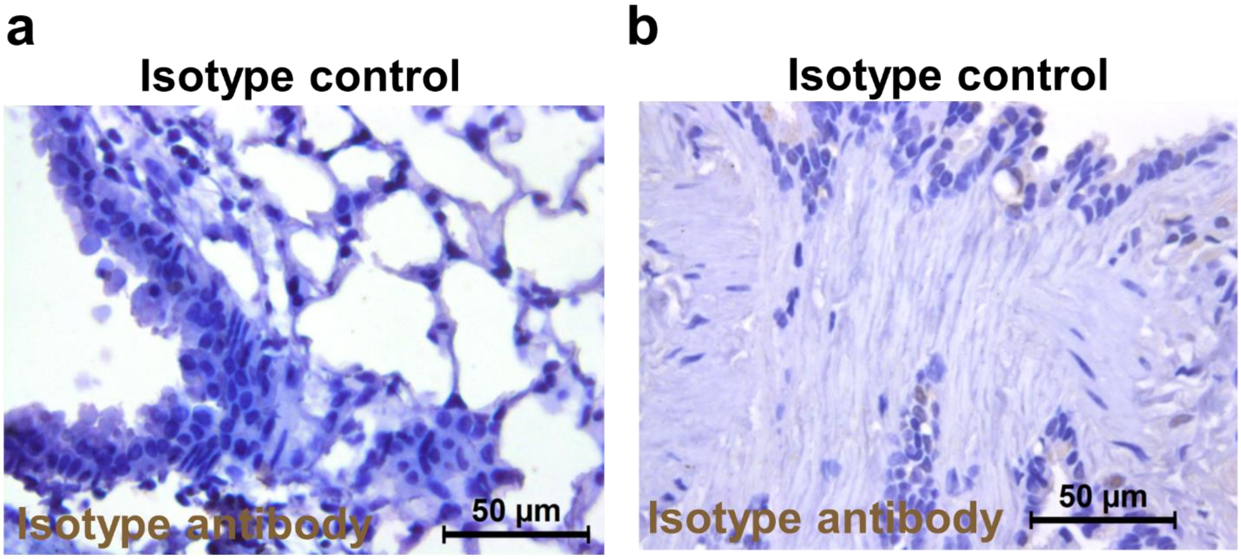

Supplementary Figure 1. Isotype control antibody immunostainings of human and mouse lung sections. **a** Representative image of lung sections from wildtype mice (n = 2) sensitized and challenged with HDM was immunostained with rat IgG and anti-rat HRP antibodies. Scale bar, 50 µm. **b** Representative images of lung sections of human asthma (n = 2) was immunostained with goat IgG and anti-goat HRP antibodies. Scale bar, 50 µm. At least two independent experiments produced similar results.

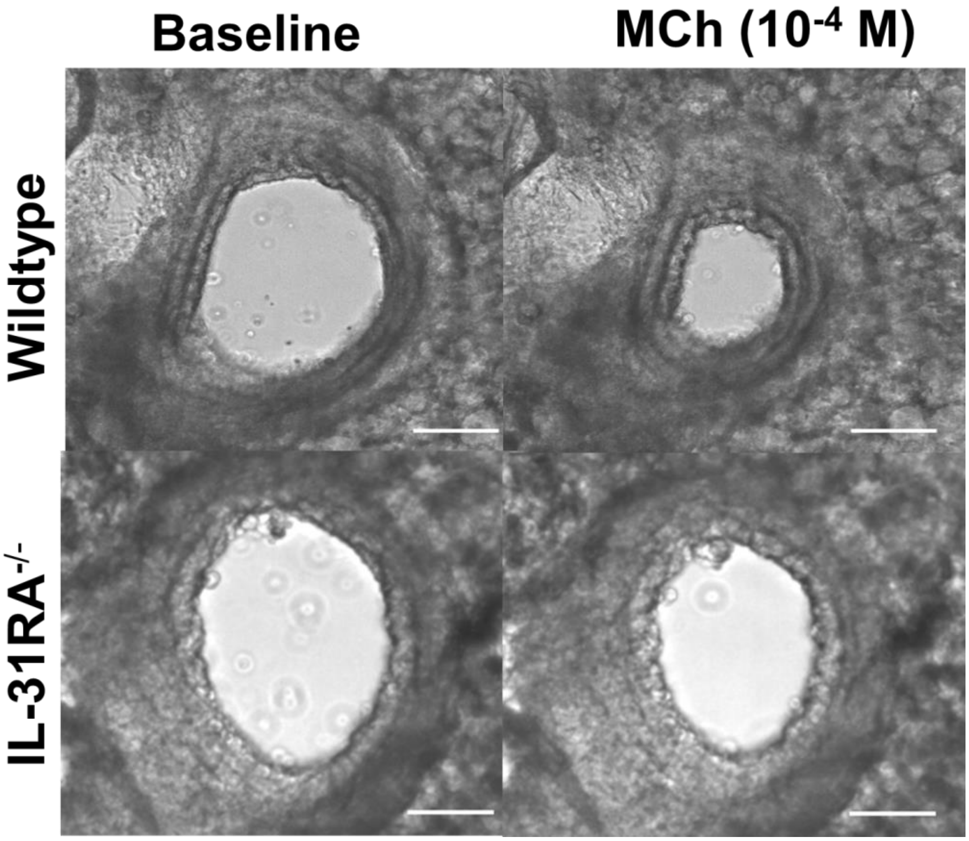

Supplementary Figure 2. Loss of IL-31RA$^{-/-}$ attenuates contraction of PCLS upon carbachol treatment. Representative images of precision cut lung sections (PCLS) from the lungs of wildtype and IL-31RA$^{-/-}$ mice treated with MCh (10^{-4} M) or prior to the treatment (baseline). Scale bar, 150 µm. At least two independent experiments produced similar results.

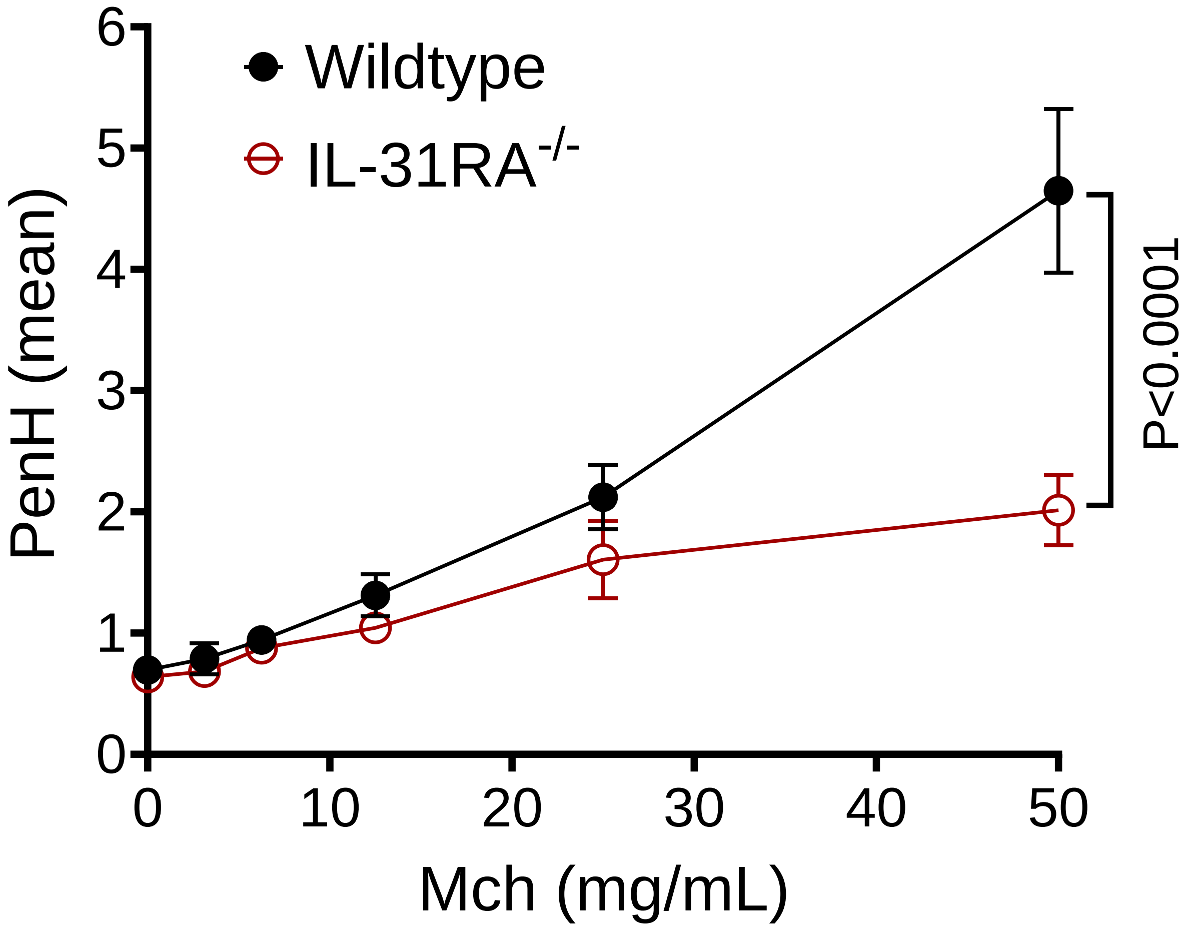

Supplementary Figure 3. The loss of IL-31RA attenuates AHR in naïve mice. Measurement of AHR with increasing doses of methacholine (MCh) in naïve wildtype (n = 18) and IL-31RA$^{-/-}$ mice (n = 19) using noninvasive whole-body plethysmography. Data are shown as mean ± SEM. A two-way ANOVA test was used. At least two independent experiments produced similar results. Source data are provided as a Source Data file.

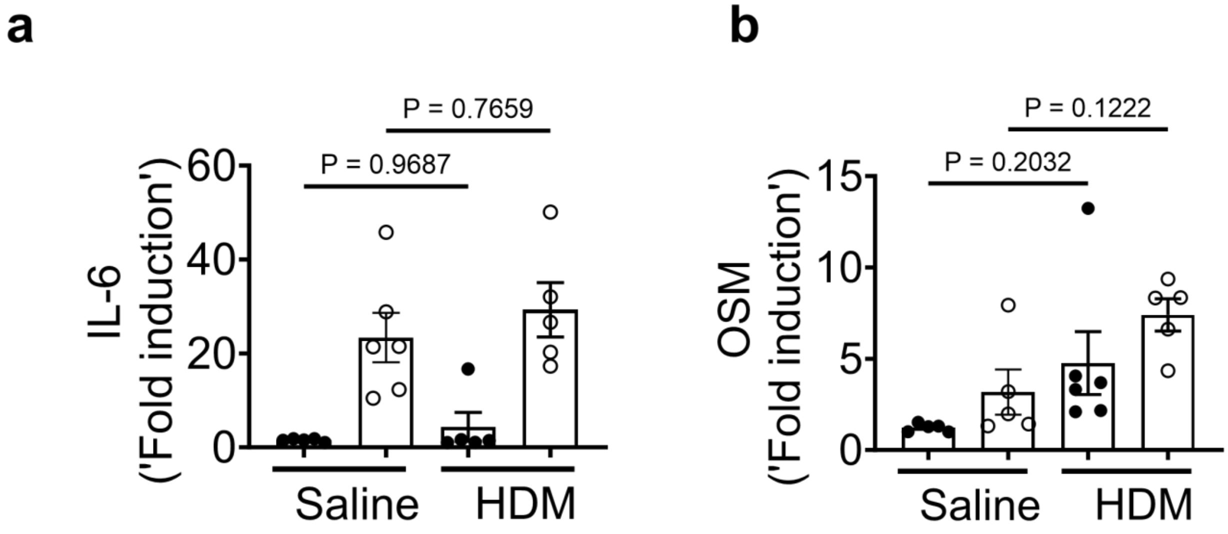

Supplementary Figure 4. Loss of IL-31RA has no effect on the expression of IL-6 and OSM in the lungs of HDM-challenged mice compared to saline-treated mice. a Quantification of IL-6 transcripts using RT-PCR in the lungs of wildtype (n = 5) and IL-31RA$^{-/-}$ (n=6) mice treated with saline or wildtype (n = 5) and IL-31RA$^{-/-}$ (n=5) mice treated with HDM. Data are shown as mean ± SEM. One-way ANOVA was used. **b** Quantification of OSM transcripts using RT-PCR in the lungs of wildtype (n = 5) and IL-31RA$^{-/-}$ (n = 5) mice treated with saline or wildtype (n = 6) and IL-31RA$^{-/-}$ (n = 5) mice treated with HDM. Data are shown as mean ± SEM. One-way ANOVA was used. No significance was found between the groups. At least two independent experiments produced similar results. Source data are provided as a Source Data file.

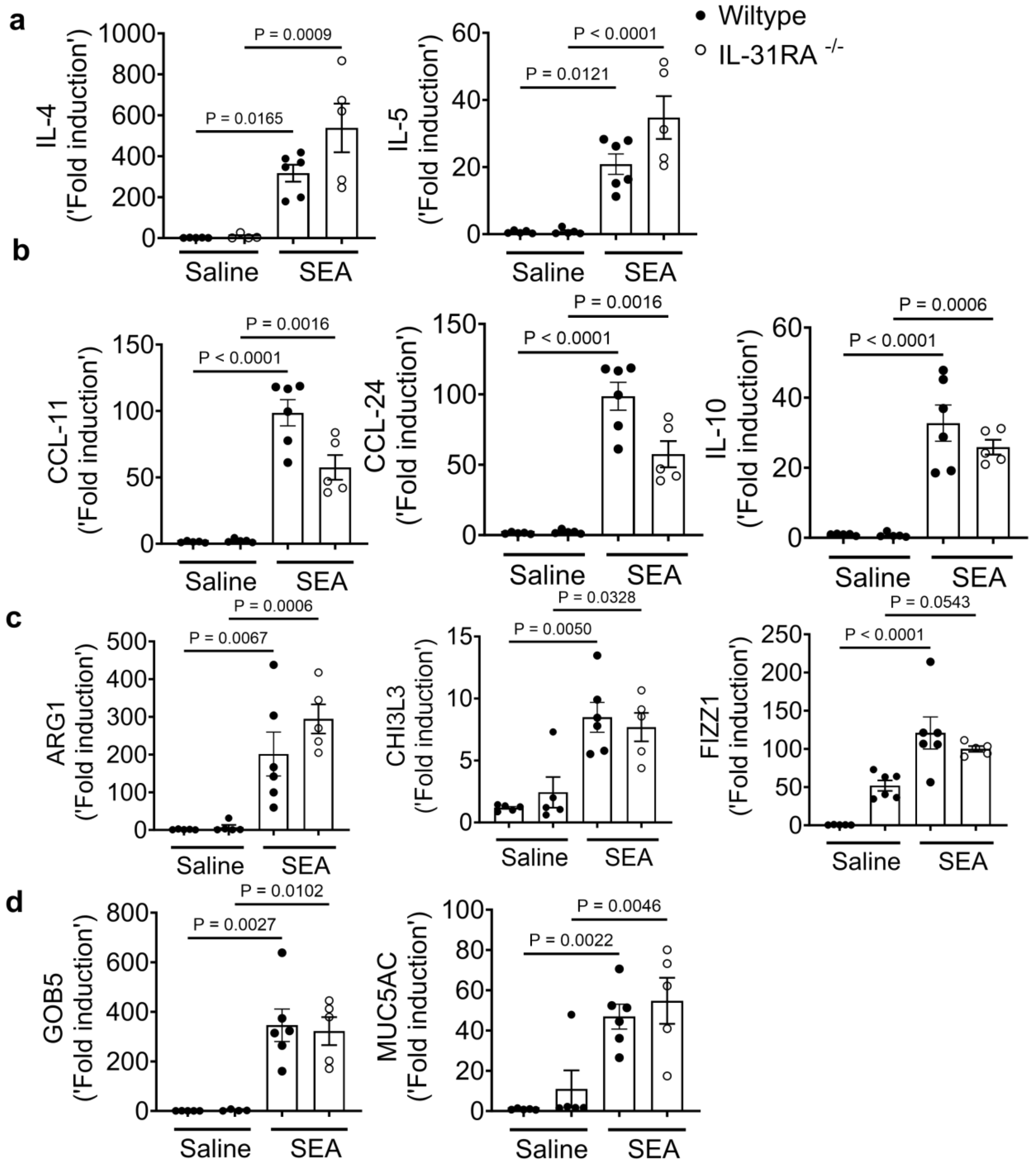

Supplementary Figure 5. Loss of IL31RA has no effect on Th2 responses and goblet cell hyperplasia during SEA-induced allergic asthma. a Quantification of IL-4, IL-5, and IL-13

using RT-PCR in the lungs of wildtype (n = 5) and IL-31RA$^{-/-}$ (n = 5) mice treated with saline or wildtype (n = 6) and IL-31RA$^{-/-}$ (n = 5) mice treated with SEA. Data are shown as mean ± SEM. Two-way ANOVA was used. **b** Quantification of CCL11, CCL24, and IL-10 using RT-PCR in the lungs of wildtype (n = 5) and IL-31RA$^{-/-}$ (n = 5) mice treated with saline or wild-type (n = 6) and IL-31RA$^{-/-}$ (n = 5) mice treated with SEA. Data are shown as mean ± SEM. Two-way ANOVA was used. **c** Quantification of Th2 response-associated genes including CHI3L3, ARG1, and FIZZ1 using RT-PCR in the lungs of wildtype (n = 5) and IL-31RA$^{-/-}$ (n = 5) mice treated with saline or wild-type (n = 6) and IL-31RA$^{-/-}$ (n = 5) mice treated with SEA. Data are shown as mean ± SEM. Two-way ANOVA was used. **d** Quantification of GOB5 and MUC5AC gene transcripts using RT-PCR in the lungs of wildtype (n = 5) and IL-31RA$^{-/-}$ (n = 5) mice treated with saline or wild-type (n = 6) and IL-31RA$^{-/-}$ (n = 5) mice treated with SEA. Data are shown as means ± SEM. Two-way ANOVA was used. At least two independent experiments produced similar results. Source data are provided as a Source Data file.

.

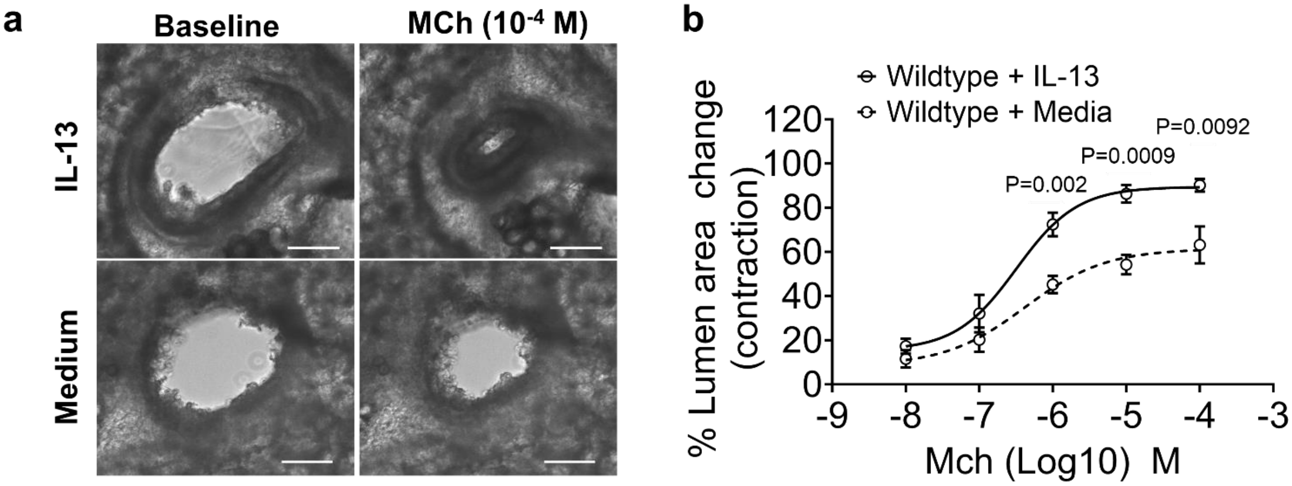

Supplementary Figure 6. IL-13 induces contraction of airways in PCLS of wild-type mice. **a** Representative images of MCh-induced airway lumen contraction in PCLS of wildtype mice treated with IL-13 (n = 6) or media (n = 8) for 24 hrs. Images were captured at 10X magnification. Scale bar, 150 µm. **b** PCLS from wildtype mice treated with IL-13 (n = 6) or media (n = 8) for 24 hrs and the percent of airway lumen area contraction with increasing doses of MCh was measured in images. Data are shown as means ± SEM. A two-way ANOVA test was used. At least two independent experiments produced similar results. Source data are provided as a Source Data file.

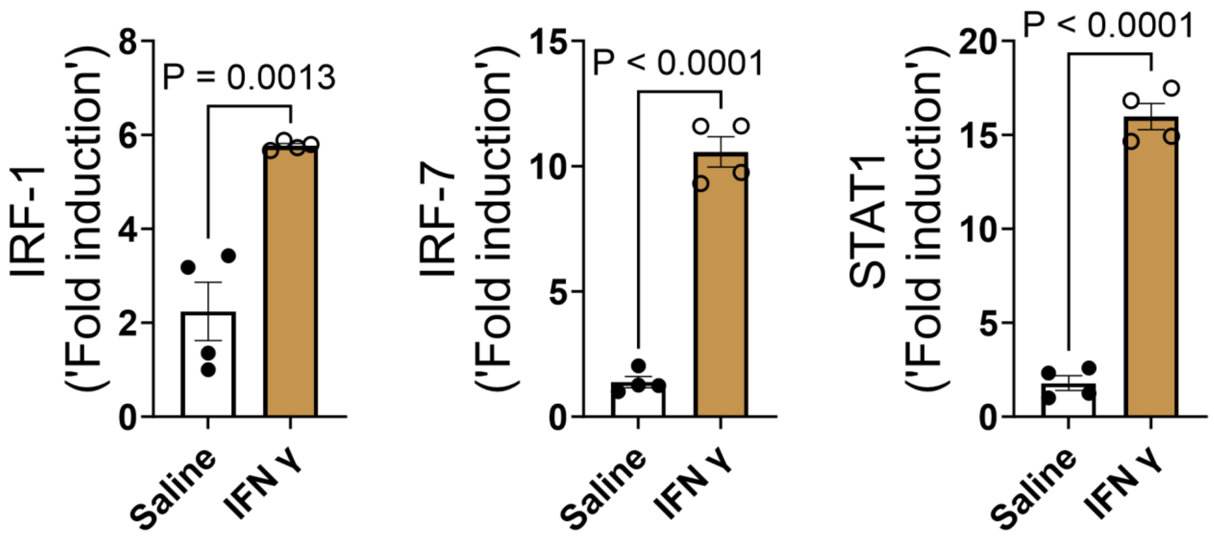

Supplementary Figure 7. Upregulation of IFNγ-induced genes in the lungs of IFNγ-treated mice. Wildtype mice were treated intratracheally with 5 µg of IFNγ (n=4) or saline (n=4) on days 0 and 6 and sacrificed on day 7. To confirm the effects of IFNγ, the expression of IFNγ-specific gene transcripts including IRF1, IRF7, and STAT1 were measured in the lungs using RT-PCR. Data are shown as means ± SEM. Unpaired *t*-test was used. At least two independent experiments produced similar results. Source data are provided as a Source Data file.

.

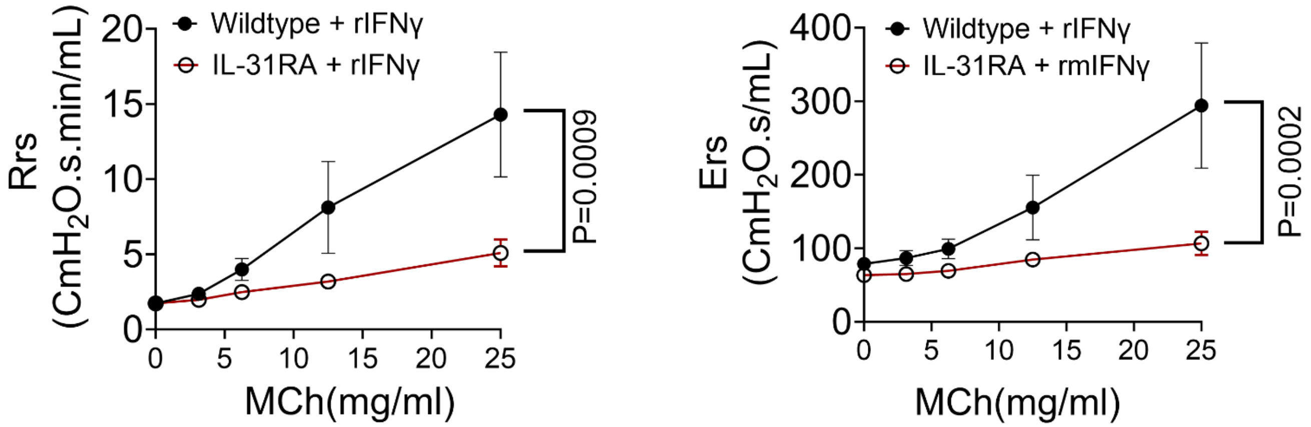

Supplementary Figure 8. IFN-γ induced responses in the wildtype and IL-31RA$^{-/-}$ mice.

Wildtype (n = 10) and IL-31RA$^{-/-}$ (n = 9) mice were treated intratracheally with IFN-γ (5 µg in 50 µl) on days 0 and 6. The methacholine-dependent increase in resistance (Rrs) and elastance (Ers) were measured using FlexiVent on day 7. Data are shown as mean ± SEM. Two-way ANOVA was used. At least two independent experiments produced similar results. Source data are provided as a Source Data file.

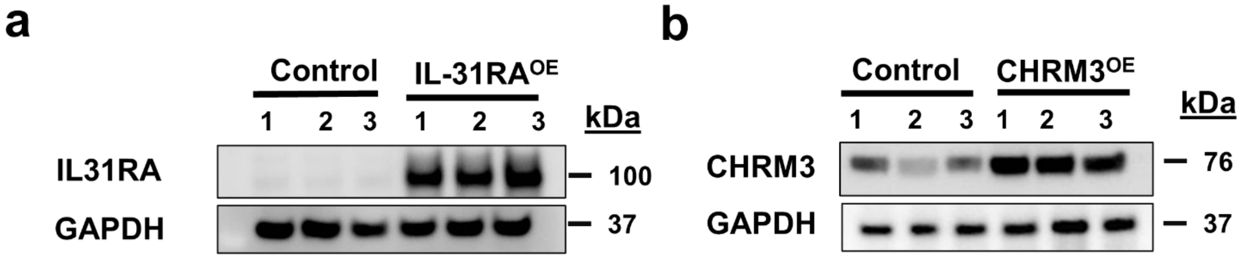

Supplementary Figure 9. Overexpression of IL-31RA and CHRM3 in HEK 293 cells. a HEK293 cells were transiently transfected with control (n=3) or overexpressing plasmid for IL-31RA (n=3) for 72 h and cell lysates were immunoblotted with antibodies against IL-31RA and GAPDH. **b** HEK293 cells were transiently transfected with control (n=3) or overexpressing plasmid for CHRM3 (n=3) for 72 h and cell lysates were immunoblotted with antibodies against CHRM3 and GAPDH. The samples were derived from the same experiment and blots were processed in parallel. At least two independent experiments produced similar results. Source data are provided as a Source Data file.

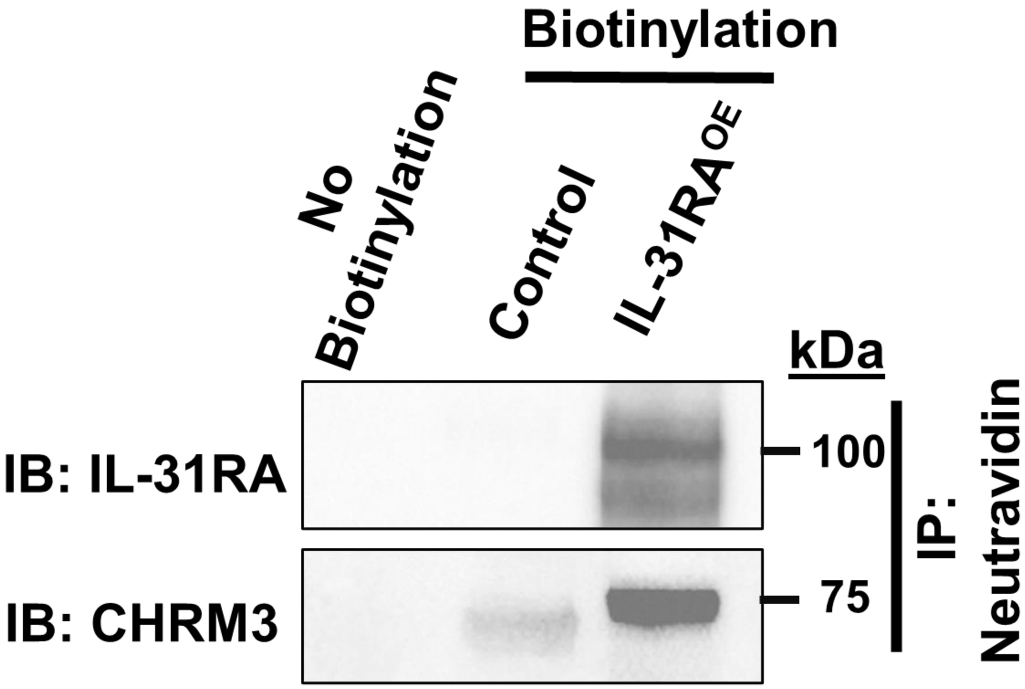

Supplementary Figure 10. Overexpression of IL-31RA promotes cell surface expression of CHRM3. HEK293 cells were transiently transfected with control plasmid or overexpressing plasmid for IL-31RA for 72 h and cell surface proteins were biotinylated and biotinylated membrane proteins from neutravidin affinity columns were immunoblotted with antibodies against IL-31RA and CHRM3. The samples were derived from the same experiment and blots were processed in parallel. At least two independent experiments produced similar results. Source data are provided as a Source Data file.

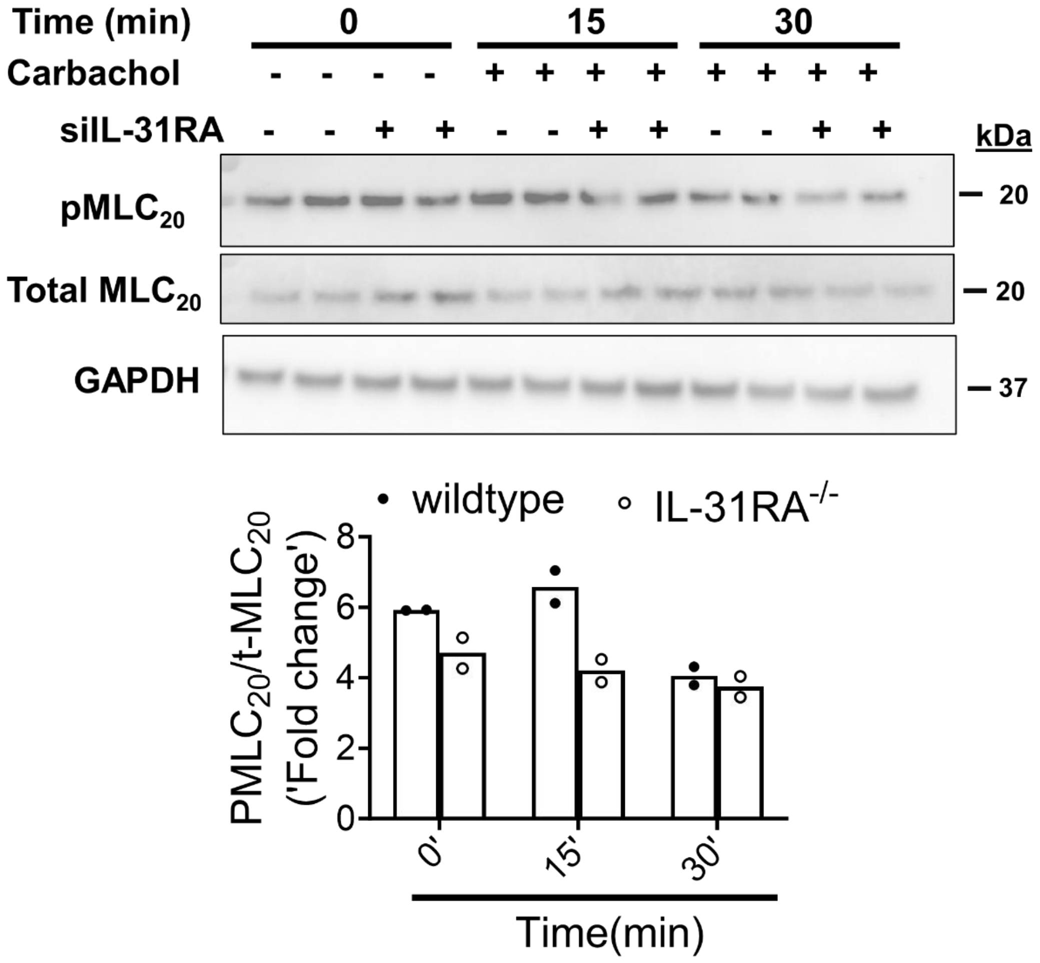

Supplementary Figure 11. Time-dependent changes in carbachol-induced phosphorylation of MLC. ASMC were isolated from the trachea of wildtype (n=2) and IL-31RA$^{-/-}$ (n=2) mice and treated with carbachol (10 µM) for 0, 15, and 30 minutes. Cell lysates were immunoblotted with antibodies against phospho-MLC total MLC, and GAPDH. The samples were derived from the same experiment and blots were processed in parallel. At least two independent experiments produced similar results. Source data are provided as a Source Data file.

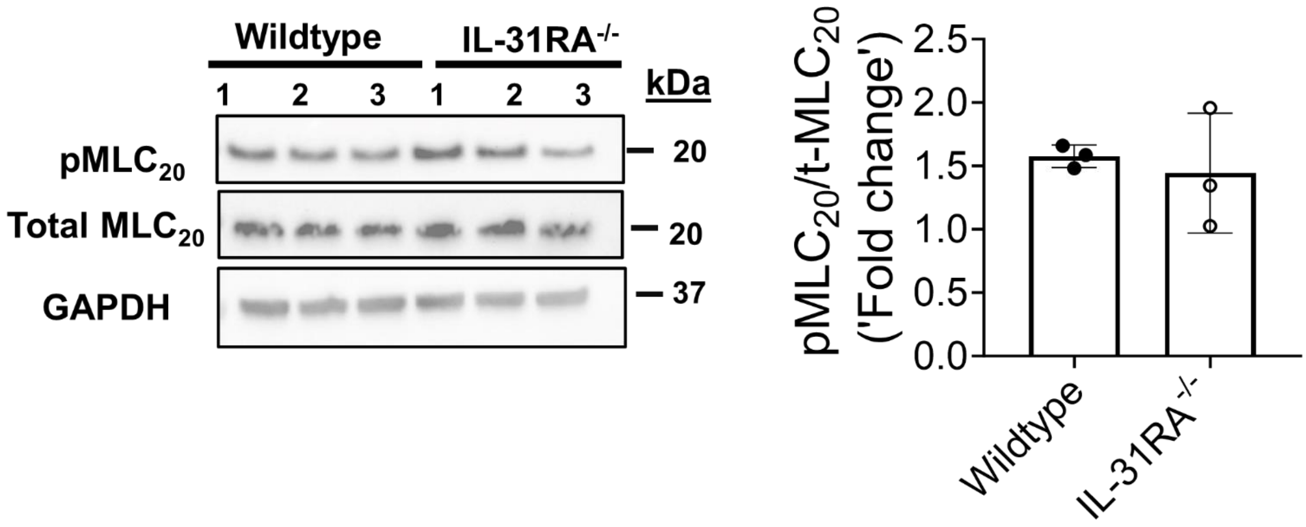

Supplementary Figure 12. Loss of IL-31RA has no effect on serotonin-induced phosphorylation of MLC. ASMC were isolated from the trachea of wildtype (n=3) and IL-31RA$^{-/-}$ (n=3) mice and treated with serotonin (100µM) for 10 min. The total cell lysates were immunoblotted with antibodies against phosphor MLC20, total MLC20, and GAPDH. An unpaired t-test was used. Data are shown as means ± SEM. The data presented is representative of two independent experiments with similar results. The samples were derived from the same experiment and blots were processed in parallel. Source data are provided as a Source Data file.

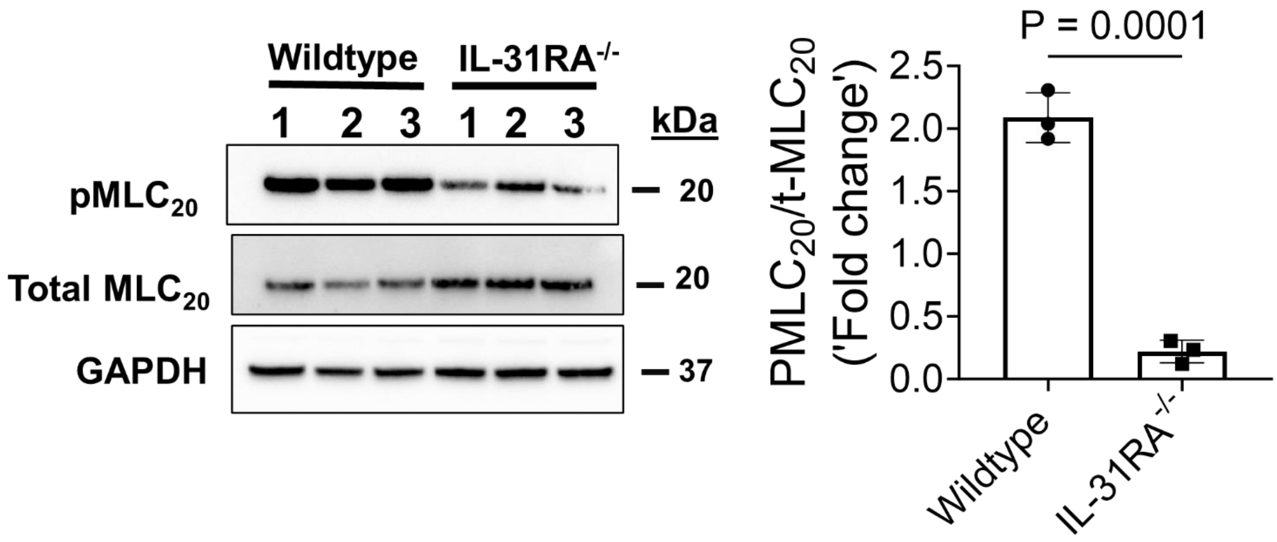

Supplementary Figure 13. Loss of IL-31RA attenuated carbachol-induced phosphorylation of MLC in intestinal SMC (ISMC). ISMCs were isolated from the large intestine of wildtype (n=3) and IL-31RA$^{-/-}$ (n=3) mice. ISMC were treated with carbachol (10 µM) for 10 min and cell lysates were immunoblotted with antibodies against phospho-MLC, total MLC, and GAPDH. Unpaired t- test was used. Data are shown as means ± SEM. The data presented is representative of two independent experiments with similar results. The samples were derived from the same experiment and blots were processed in parallel. Source data are provided as a Source Data file.

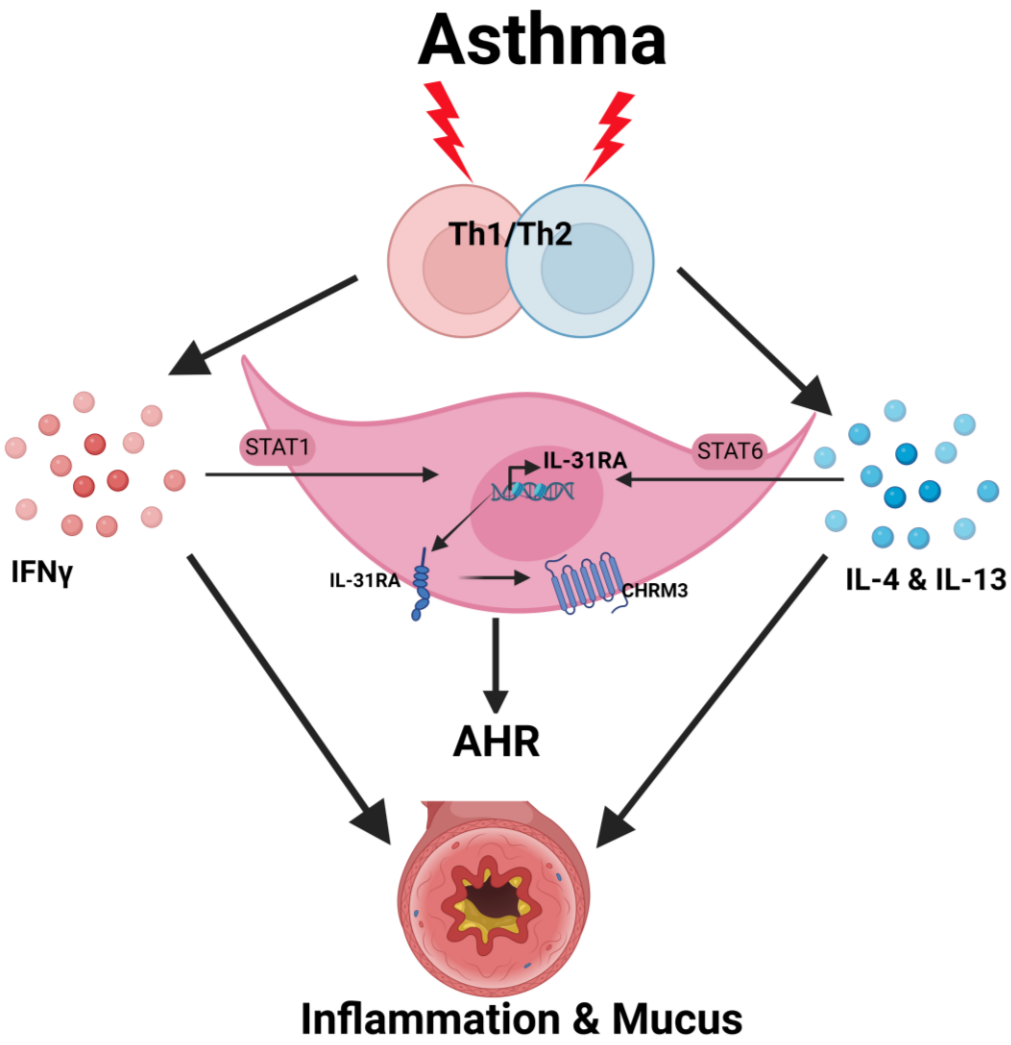

Supplementary Figure 14. Schema showing mechanisms underlying Th1/Th2-cytokine driven AHR in asthma. The loss of IL-31RA was sufficient to attenuate CHRM3 expression, calcium signaling, and contractility of ASMC and improved AHR in animal models in vivo. However, Th2 responses, inflammation, and goblet cell hyperplasia has remained unchanged with IL-31RA deficiency during allergen-induced asthma. IL-31RA, Interleukin 31 receptor alpha; CHRM3, Cholinergic receptor muscarinic 3; IL-4, Interleukin 4; IL-13, interleukin 13; IFNγ, Interferon gamma; STAT1, Signal transducer and activator of transcription 1; STAT6, Signal transducer and activator of transcription 6. Image was created with Biorender.com.

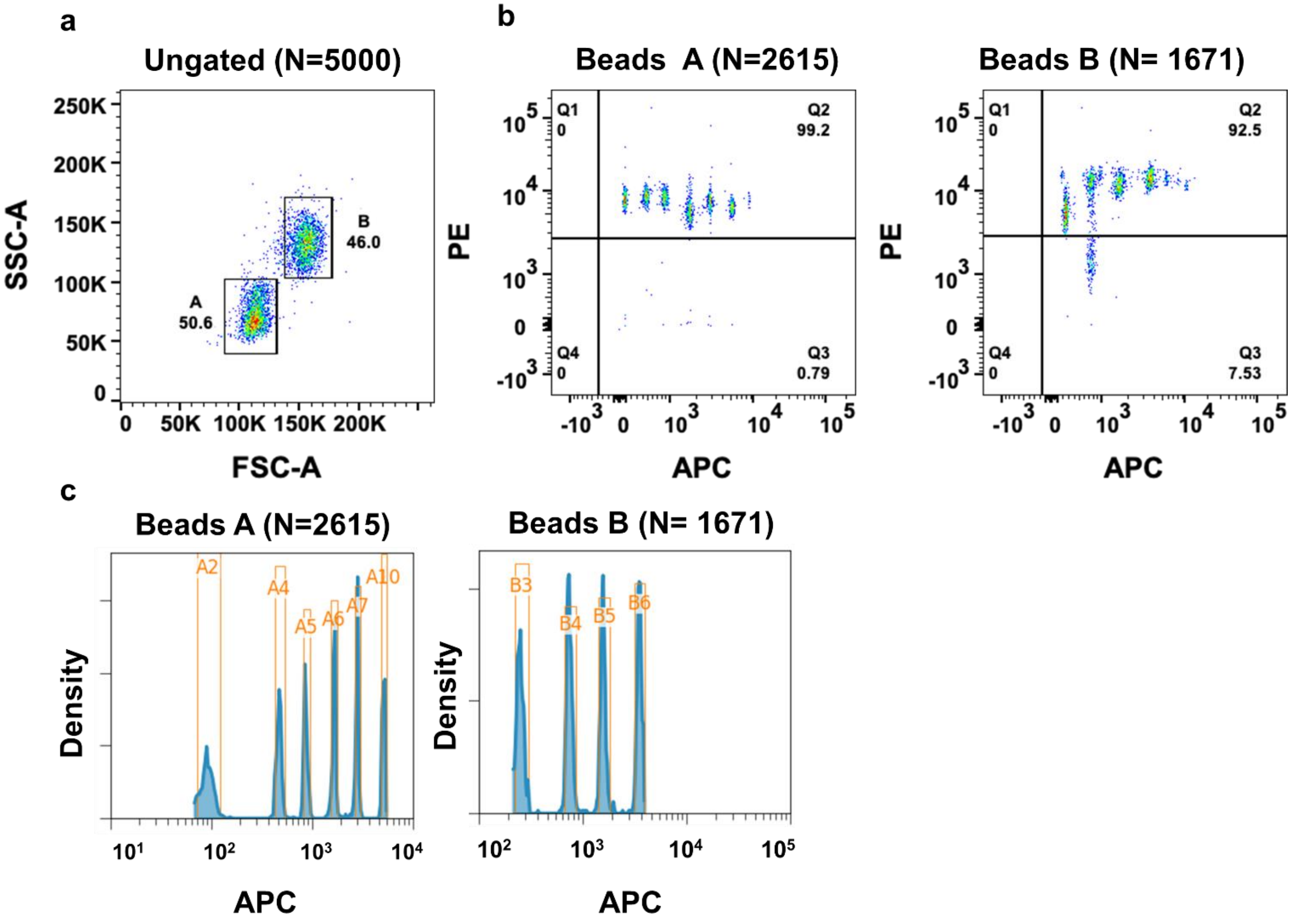

Supplementary Figure 15. Gating strategy of BAL cytokines and chemokines analysis using flow cytometry. BAL Cytokine and chemokine concentrations were determined using Flow cytometry-based bead assay (LENGENDplex). **a** The chemokines and cytokine binding beads were gated based on SSC and FCS (linear mode) to identify beads A and beads B. **b** Beads A and beads B were gated on APC and PE in log mode. **c** The concentration of cytokines and chemokines was determined using LEGENDplex data Analysis Software according to the manufacturer instructions. At least two independent experiments produced similar results. Source data are provided as a Source Data file.

Supplementary Table 1. The details of antibodies including vendor source, dilution, catalog number, and application used are listed below.

Antibody	Supplier	Catalog #	Lot	Dilution	Application	Species reactivity
CHRM3	Abcam	ab126168	GR3461422-1	1 in 1000	Western blotting	Polyclonal: human and mouse
β-actin	Santacruz		K2706	1 in 1000	Western blotting	Monoclonal: mouse, rat, and human
GAPDH	Bethyl	A300-643A	2	2 in 1000	Western blotting	Polyclonal: human and mouse
hIL-31RA	R&D	AF2769	VNG0122021	1 in 1000, 15µg/ml	Western blotting and IHC	Polyclonal goat IgG: human
ITGB1	Cell signaling technology	4706	6	1 in 1000	Western blotting	Monoclonal: mouse
mIL-31RA (gift)	Bristol myers squibb			2µg/ml	Immunohistochemistry	Monoclonal: mouse
myosin light chain 2	Cell signaling technology	3672S	7	1 in 1000	Western blotting	Polyclonal: human , mouse, and rat
p-myosin Light chain 2(T18/S19)	Cell signaling technology	3674S	6	1 in 1000	Western blotting	Polyclonal: human and mouse
ANTI-FLAG®	Sigma	F7425-.2MG	113663	15µg/ml	Immunoprecipitation	Polyclonal
Anti-FLAG®-Peroxidase	Sigma	H7425-1VL	160280	1 in 2000	Immunoprecipitation	Rabbit
Biotinylated Anti-Goat IgG (H+L)	Vector	BA5000	ZA0425	1 in 200	Immunohistochemistry	Goat
Anti-rabbit IgG, HRP-linked antibody	Cell signaling technology	7074P2	32	1 in 10000	Western blotting	Rabbit
Anti-mouse IgG, HRP-linked Antibody	Cell signaling technology	7076S	34	1 in 20000	Western blotting	Mouse
Biotinylated Anti-rat IgG (H+L)	Vector	BA4001	Y0809	1 in 200	Immunohistochemistry	Rat
Goat IgG	R&D	AB108C	ES4521111	15µg/ml	Immunohistochemistry	Goat
Rabbit IgG	Vector	I-1000	2H1201	2µg/ml	Immunoprecipitation	Rabbit
Cholera toxin b Alexa 488	Thermo Fischer	c34775	2018246	1 in 100	Immunoflurescence	

Supplementary Table 2. The sequences of gene-specific RT-PCR primers and siRNA are listed below.

**Gene ID**	**FORWARD**	**REVERSE**
hIL-31	GCCCAGCCGCCAAAC	GCTGTCTGATTGTCTTGAGATATGC
hIL-31RA	TAGTACCAGATCATCTGTGT	TTAGACTTCTCCCTTGGTGTGC
hβ ACTIN	CCA ACC GCG AGA AGA TGA	CCA GAG GCG TAC AGG GAT AG
mARG1	GGAAAGCCAATGAAGAGCTG	GCTTCCAACTGCCAGACTGT
mCCL11	AGAGCTCCACAGCGCTTCT	GCAGGAAGTTGGGATGGA
mCCL24	GCAGCATCTGTCCCAAGG	GCAGCTTGGGGTCAGTACA
mCHIL3L	GTAGCACATCAGCTGGTAGGA	AAAGGCAAAACTGTTAGCCAAGG
mFIZZ1	CCCTCCACTGTAACGAAGACTC	CACACCCAGTAGCAGTCATCC
mGOB5	AGGAAAACCCCAAGCAGTG	GCACCGACGAACTTGATTTT
mHPRT	GCCCTTGACTATAATGAGTACTTCAGG	TTCAACTTGCGCTCATCTTAGG
mIFNγ	AGAGCCAGATTATCTCTTTCTACCTCAG	CCTTTTTCGCCTTGCTGTTG
mIL-4	ACGAGGTCACAGGAGAAGGGA	AGCCCTACAGACGAGCTCACTC
mIL-5	TGACAAGCAATGAGACGATGAGG	ACCCCCACGGACAGTTTGATTC
mIL-6	GCTACCAAACTGGATATAATCAGGA	CCAGGTAGCTATGGTACTCCAGAA
mIL-10	CAGAGCCACATGCTCCTAGA	GTCCAGCTGGTCCTTTGTTT
mIL-13	CCTCTGACCCTTAAGGAGCTTAT	CGTTGCACAGGGGAGTCTT
mIL-17	CAGGGAGAGCTTCATCTGTGT	GCTGAGCTTTGAGGGATGAT
mIL-31	TCTTACCGTCGCCATGATCT	GCACCGAAGGACAAGCTG
mIL-31RA	CAGAATTCTCCACAGGTCCAG	TGGAGCAAAGAAGAAACCAGA
mIRF-1	GTTGTTTACAGCGTGTGGCTT	AGCCAGCAAAAGACTCCCAT
mIRF-7	TCCAGTTGATCCGCATAAGGT	CTTCCCTATTTTCCGTGGCTG
mMUC4	CAGGCAAGGTTGAGGTATCC	CATGCATAAGAAAAGGCGGCA
mMUC5AC	ACTTCAACGGCAGTCCAAAA	CTCAAGGGGTGTCAGCCTAA
mOSM	TGCTCCAACTCTTCCTCTCAG	CAGGTTTTGGAGGCGGATA

mSOCS3	ATTTCGCTTCGGGACTAGC	AACTTGCTGTGGGTGACCAT
mSTAT1	CCCCTGAAGTATCTGTACCCCA	CTCAGGCACTCACCTTCCTTT
mTNFα	GCCTCTTCTCATTCCTGCTTGT	GGCCATTTGGGAACTTCTCAT
siIL-31RA<br>**Assay id:**<br>HSS134470	**Sense strand:**<br>CCAGTGACAAGTTGGTGATTGACAA	
