# Supplementary material for: Interleukin 31 receptor α promotes smooth muscle cell contraction and airway hyperresponsiveness in asthma
Source: Nat Commun. 2023 Dec 11;14:8207. doi: 10.1038/s41467-023-44040-1 (PMC10713652; doi:10.1038/s41467-023-44040-1)
Supplement: Supplementary file 3 — Reporting Summary [file 41467_2023_44040_MOESM3_ESM.pdf]

nature portfolio

Corresponding author(s): Satish Madala

Last updated by author(s): Oct 21, 2023

Reporting Summary

Nature Portfolio wishes to improve the reproducibility of the work that we publish. This form provides structure for consistency and transparency in reporting. For further information on Nature Portfolio policies, see our Editorial Policies and the Editorial Policy Checklist.

Statistics

For all statistical analyses, confirm that the following items are present in the figure legend, table legend, main text, or Methods section.

n/a	Confirmed	
☐	☒	The exact sample size (*n*) for each experimental group/condition, given as a discrete number and unit of measurement
☐	☒	A statement on whether measurements were taken from distinct samples or whether the same sample was measured repeatedly
☐	☒	The statistical test(s) used AND whether they are one- or two-sided<br>*Only common tests should be described solely by name; describe more complex techniques in the Methods section.*
☐	☒	A description of all covariates tested
☐	☒	A description of any assumptions or corrections, such as tests of normality and adjustment for multiple comparisons
☐	☒	A full description of the statistical parameters including central tendency (e.g. means) or other basic estimates (e.g. regression coefficient) AND variation (e.g. standard deviation) or associated estimates of uncertainty (e.g. confidence intervals)
☐	☒	For null hypothesis testing, the test statistic (e.g. F, t, r) with confidence intervals, effect sizes, degrees of freedom and P value noted<br>*Give P values as exact values whenever suitable.*
☒	☐	For Bayesian analysis, information on the choice of priors and Markov chain Monte Carlo settings
☒	☐	For hierarchical and complex designs, identification of the appropriate level for tests and full reporting of outcomes
☒	☐	Estimates of effect sizes (e.g. Cohen's *d*, Pearson's *r*), indicating how they were calculated

Our web collection on statistics for biologists contains articles on many of the points above.

Software and code

Policy information about availability of computer code

Data collection	Airway hyperresponsiveness was measured using SCIREQ,; Calcium assays were performed on softmax pro 11; Immunoblots were collected on Biorad ChemiDOC MP system and analyzed on biorad Image lab software 6.1; RT-PCR were analyzed on CFX Maestro software version 4.0; Immunofluorescence data were analyzed on the NIS elements; cytokines concentrations were analyzed using LegendPLEX software.
Data analysis	GraphPad Prism (v.10.0.2), Imaris (v.10.0)

For manuscripts utilizing custom algorithms or software that are central to the research but not yet described in published literature, software must be made available to editors and reviewers. We strongly encourage code deposition in a community repository (e.g. GitHub). See the Nature Portfolio guidelines for submitting code & software for further information.

Data

Policy information about availability of data

All manuscripts must include a data availability statement. This statement should provide the following information, where applicable:

- Accession codes, unique identifiers, or web links for publicly available datasets
- A description of any restrictions on data availability
- For clinical datasets or third party data, please ensure that the statement adheres to our policy

Data sets will be available and no restrictions on data availability

Research involving human participants, their data, or biological material

Policy information about studies with human participants or human data. See also policy information about sex, gender (identity/presentation), and sexual orientation and race, ethnicity and racism.

Reporting on sex and gender	De-identified pediatric lung tissue samples were used for immunohistochemistry staining of IL-31RA. The slides used for immunostaining include lung samples from asthma (n=8; 6 males and 2 females) and healthy controls (n=8; 5 males and 3 females).
Reporting on race, ethnicity, or other socially relevant groupings	n/a
Population characteristics	n/a
Recruitment	biopsies were collected
Ethics oversight	n/a

Note that full information on the approval of the study protocol must also be provided in the manuscript.

Field-specific reporting

Please select the one below that is the best fit for your research. If you are not sure, read the appropriate sections before making your selection.

☒ Life sciences ☐ Behavioural & social sciences ☐ Ecological, evolutionary & environmental sciences

For a reference copy of the document with all sections, see nature.com/documents/nr-reporting-summary-flat.pdf

Life sciences study design

All studies must disclose on these points even when the disclosure is negative.

Sample size	The power analysis method for estimating sample size for animal studies depends on detecting significant differences between saline and allergen treatment, using independent two-sample t-tests at alpha = 0.05, for 90% power.
Data exclusions	none
Replication	all experiments were repeated multiple times with similar results
Randomization	for animal studies we used equal number of males and females with similar age.
Blinding	For cell counting slides were blinded and counted

Reporting for specific materials, systems and methods

We require information from authors about some types of materials, experimental systems and methods used in many studies. Here, indicate whether each material, system or method listed is relevant to your study. If you are not sure if a list item applies to your research, read the appropriate section before selecting a response.

Materials & experimental systems

n/a	Involved in the study	
☐	☒	Antibodies
☐	☒	Eukaryotic cell lines
☒	☐	Palaeontology and archaeology
☐	☒	Animals and other organisms
☒	☐	Clinical data
☒	☐	Dual use research of concern
☒	☐	Plants

Methods

n/a	Involved in the study	
☒	☐	ChIP-seq
☐	☒	Flow cytometry
☒	☐	MRI-based neuroimaging

Antibodies

Antibodies used	primary antibodies: anti-CHRM3 abcam; ab126168,GR3461422-1; anti-hIL-31RA ; R&D ;AF2769; VNG0122021; anti-p-myosin Light chain 2(T18/S19) ;cell signaling technology ;3674S ;Lot: 6; anti-myosin light chain 2 ; cell signaling technology ;3672S ; lot :7; GAPDH

	Bethyl ; A300-643A Lot: 2 ; ANTI-FLAG® ,sigma, F7425.2MG, 113663;<br><br>Secondary antibodies:<br>Biotinylated Anti-goat IgG(H+L) ,vector, BA5000,ZA0425; Biotinylated anti-rat IgG(H+L),vector,BA4001,Y0809;Anti -Flag peroxidase, sigma,H7425-1vL,160280; Anti -rabbitIgG HRP-linked,CST, 7074P2 ,32;Anti mouse IgG HRP linked, 7076S ,34,Goat IgG ,R7D ,AB108, Rabbit IgG ,vector,I-1000,2H1201.
Validation	All antibodies were validated by commercial suppliers for immunoblotting and/or immunofluorescence. Detailed information can be found on commercial websites.<br>chrm3:https://www.abcam.com/products/primary-antibodies/muscarinic-acetylcholine-receptor-m3chrm3-antibody-ab126168.html<br>GAPDH:https://www.biomol.com/products/antibodies/primary-antibodies/general/anti-gapdh-a300-643a-t<br>IL-31RA:https://www.rndsystems.com/products/human-il-31ra-antibody_af2769<br>ITGB1:https://www.cellsignal.com/products/primary-antibodies/integrin-b1-antibody/4706<br>myosin light chain 2 :https://www.cellsignal.com/products/primary-antibodies/myosin-light-chain-2-antibody/3672<br>p-myosinLight chain 2(T18/S19) :https://www.cellsignal.com/products/primary-antibodies/phospho-myosin-light-chain-2-thr18-ser19-antibody/3674<br>ANTI-FLAG®: https://www.sigmaaldrich.com/IN/en/product/sigma/f7425<br>Anti-FLAG®-Peroxidase :https://www.sigmaaldrich.com/IN/en/product/sigma/h7425<br>Biotinylated Anti-goat IgG(H+L):https://vectorlabs.com/products/biotinylated-rabbit-anti-goat-igg/<br>Biotinylated Anti-rat IgG(H+L):https://vectorlabs.com/products/biotinylated-rabbit-anti-rat-igg-mouse-adsorbed/<br>Anti-rabbit IgG, HRP-linked Antibody:https://www.cellsignal.com/products/secondary-antibodies/anti-rabbit-igg-hrp-linked-antibody/7074<br>Anti-mouse IgG, HRP-linked Antibody: https://www.cellsignal.com/products/secondary-antibodies/anti-mouse-igg-hrp-linked-antibody/7076<br>Goat IgG:https://www.rndsystems.com/products/normal-goat-igg-control_ab-108-c<br>Rabbit IgG:https://vectorlabs.com/products/rabbit-igg/

Eukaryotic cell lines

Policy information about cell lines and Sex and Gender in Research

Cell line source(s)	Human bronchial smooth muscle cells were obtained from Lonza (Wakersville, MD, USA), Material no: 00194850 ,batch no: 0000195154; 293 [HEK-293] was obtained from ATCC (CRL1573).
Authentication	none of the cell lines used were authenticated.
Mycoplasma contamination	All cell lines were tested for mycoplasma contamination
Commonly misidentified lines<br>(See ICLAC register)	No commonly misidentified cell lines were used

Animals and other research organisms

Policy information about studies involving animals; ARRIVE guidelines recommended for reporting animal research, and Sex and Gender in Research

Laboratory animals	Wild-type and interleukin receptor IL-31RA-/- mice with a C57BL/6 background of 12–16 weeks of age were used for experiments
Wild animals	The study did not involve wild animals
Reporting on sex	Age-matched male and female mice of 12–16 weeks of age were used for experiments with at least two repeats.
Field-collected samples	The study did not involve field collected samples
Ethics oversight	All mice were housed under specific pathogen-free conditions at the Cincinnati Children's Hospital Medical Center, a medical facility approved by American Association for the Accreditation of Laboratory Animal Care. All experimental procedures were approved by Animal Care and Use Committee of Cincinnati Children's Hospital Medical Center.

Note that full information on the approval of the study protocol must also be provided in the manuscript.

Plants

Seed stocks: n/a

Novel plant genotypes: n/a

Authentication: n/a

Flow Cytometry

Plots

Confirm that:

☒ The axis labels state the marker and fluorochrome used (e.g. CD4-FITC).

☒ The axis scales are clearly visible. Include numbers along axes only for bottom left plot of group (a 'group' is an analysis of identical markers).

☒ All plots are contour plots with outliers or pseudocolor plots.

☐ A numerical value for number of cells or percentage (with statistics) is provided.

Methodology

Sample preparation: Beads-based chemokine and cytokine panel flow cytometry
A custom LEGENDplexTM mouse Panel 761 kit (bead-based assay including chemokines and cytokines of interest) was used (Biolegend, item # 9000001861, lot number # B394351). The BAL fluid (BALF) was used to measure the concentration of cytokines and chemokines of interest. BALF was collected/prepared as follows: Soon after mice were anesthetized, the lungs were lavaged twice with 1 mL of ice-cold and sterile PBS using a 1 mL insulin syringe through a tracheal catheter. BAL fluid (BALF) was centrifuged for 5 min at 250 × g at 4 °C. Cell pellets containing BAL cells were pooled and resuspended in 500 µL of PBS. The supernatant from the first BALF aspiration was stored at -80 °C and used for cytokine and chemokine measurements. The BALF samples were diluted 1:2 in PBS and performed bead binding and PE staining according to the manufacturer's manual.

No flow cytometry analysis was performed on any cell population in this manuscript, except for the cytokines and chemokines detection in BALF of mice using LegendPlex from Biolegend. A number value for number of cells do not apply for fluorescence bead based analysis.

Instrument: BD FACS LSR Fortessa analyzer (BD)

Software: FACSDiva software v8.0 (BD), LEGENDplex v8.0 data Analysis Software

Cell population abundance: Not applicable: No cell analysis or cell sorting was performed

Gating strategy: Chemokine and cytokine panel gating strategy: The chemokines/ cytokines binding beads were gated based on SSC and FCS (linear mode) to determine beads A and beads B. Then both beads A and beads B were gated on APC and PE in log mode. The result gatings and calculayions were performed on LEGENDplex data Analysis Software according to the manufacturer's manual

☒ Tick this box to confirm that a figure exemplifying the gating strategy is provided in the Supplementary Information.
